# Pancreatitis Prevention in Tabuk City, Kingdom of Saudi Arabia: Evaluating Public Knowledge and Raising Awareness of Risk Factors and Symptoms

**DOI:** 10.7759/cureus.47069

**Published:** 2023-10-15

**Authors:** Yousef Alalawi, Abdulrahman A Daghreeri, Abdulaziz I Alkhudairy, Abdullah A Ali, Mohammed N Alahmari, Faisal N Alahmari, Roaa G Khan, Afnan Al-Zayed, Mohammed Alamri

**Affiliations:** 1 Surgery, King Salman Armed Forces Hospital, Tabuk, SAU; 2 Medicine and Surgery, Jazan University, Jazan, SAU; 3 Medicine, King Saud Bin Abdulaziz University for Health Sciences, Riyadh, SAU; 4 Medicine and Surgery, King Khalid University, Abha, SAU; 5 Surgery, University of Tabuk, Tabuk, SAU; 6 Medicine, King Saud bin Abdulaziz University for Health Sciences, Riyadh, SAU; 7 College of Medicine, Faculty of Medicine, King Abdulaziz University, Jeddah, SAU

**Keywords:** saudi arabia, awareness, complications, knowledge, pancreatitis

## Abstract

Introduction: Pancreatitis is an inflammation of the pancreas. The pancreas is a flat, elongated gland situated in the upper abdomen, beyond the stomach. It produces digestive enzymes and hormones that regulate glucose absorption in the body. Pancreatitis can be acute, developing rapidly and lasting for several days, or chronic, persisting over an extended period and affecting specific individuals. While treatment can improve mild cases of pancreatitis, severe cases can be fatal.

Method: This study utilizes a cross-sectional survey design with 549 participants, allowing data collection from a representative sample of Tabuk City's adult population.

Results: The participants' knowledge about the risk factors and symptoms of pancreatitis was inadequate. Among those who did not receive any information about pancreatitis and its risk factors, the count was 352 (64.1%). On the other hand, there was increased awareness of pancreatitis and its risk factors, which would lead to early detection and prevention. A total of 483 participants (88%) expressed adequate agreement, and 305 participants (55.6%) demonstrated an adequate response regarding seeking medical attention if they experienced any symptoms of pancreatitis.

Conclusion: Our findings revealed a lack of knowledge about the risk factors and symptoms of pancreatitis. Furthermore, there was inadequate awareness regarding governmental initiatives or programs that support access to pancreatitis knowledge and awareness in Tabuk City.

## Introduction

Pancreatitis, an inflammatory condition of the pancreas, is primarily caused by factors such as gallstones and excessive alcohol intake rather than infectious diseases. The pathogenesis and development of the disease may be influenced by complex gene-environment interactions, or they may impact them. The disease's pathogenesis and development are influenced by complex gene-environment interactions, leading to the concept of a disease continuum, replacing the notion of distinct disease entities. Notably, 30% of individuals with acute pancreatitis progress to a chronic condition, often involving recurrent pancreatitis in subsequent years [1].

Pancreatitis, both acute and chronic, is a prevalent condition affecting individuals globally. These diseases pose significant public health concerns due to their high mortality rates and the substantial financial burden they place on healthcare systems in numerous nations. Acute pancreatitis (AP), initially deemed self-limiting, has shown an increasing occurrence in Western countries, ranging from 5 to 10 cases per 100,000 to 70 to 80 cases per 100,000 [2].

Recurrent AP (RAP) serves as an intermediate stage between AP and CP, as proposed by an evolving disease theory. While most studies on this topic have been conducted in Western nations, some have delved into the natural progression of pancreatitis, including the risk and preventive factors contributing to the transition from AP to RAP and CP. Notably, smoking, in addition to alcohol consumption, stands out as a significant risk factor for CP and is believed to expedite the development of alcoholic CP [3,4].

Gallstones, along with alcoholism, constitute one of the most prevalent causes of acute pancreatitis, accounting for the underlying etiology in 30%-50% of cases. Cross-sectional studies of pancreatitis patients reveal that the biliary etiology of pancreatitis is more prevalent in women, primarily due to the higher incidence of gallbladder stones among women than men [5]. Gallstones can be found in up to 20% of adults and predispose individuals to either gallbladder or biliary tract stones. Remarkably, while 75% of individuals with gallbladder stones remain asymptomatic, 8% of those with gallstones eventually suffer from acute pancreatitis [6].

Regardless of their original location, acute pancreatitis often serves as the initial indication of biliary stones. Although most patients with biliary acute pancreatitis recover fully after a moderate edematous pancreatitis episode, 15%-30% of individuals experience severe necrotizing pancreatitis, requiring immediate care and multiple treatment approaches. Gallstones that have been affected in the duodenal papilla and block the pancreatic duct's outflow cause acute pancreatitis. This causes a rise in pancreatic pressure, sometimes only momentarily, but also damages acinar cells and starts the disease [7,8].

Fever, a marker of infection or inflammation; hypovolemia; Cullen's and Grey-Turner's signs, which indicate hemorrhagic pancreatitis; tetany due to hypocalcemia; and fulminant pancreatitis are all warning signs of pancreatitis. An acute pancreatitis diagnosis must include a fever. In the first week, inflammatory cytokines are responsible for mediating it. Infection of necrotic tissues might cause fever to develop in the second or third week. Pleural effusion can also happen in some patients [9]. The diagnosis of acute pancreatitis is based on typical abdominal discomfort, a threefold increase in blood amylase or lipase levels, and positive cross-sectional abdominal imaging results [10,11].

Patients who develop pancreatitis may face severe health consequences, including high morbidity and mortality rates. Acute pancreatitis can result in severe complications such as pancreatic necrosis, infections in nearby tissues, abscess formation, sepsis, and multiple organ failure, accompanied by intense abdominal pain. Chronic pancreatitis can cause the gradual deterioration of pancreatic tissue, leading to the loss of both the exocrine and endocrine functions of the pancreas. This deterioration can result in dietary deficiencies, diabetes mellitus, and nutrient malabsorption [12]. The literature on knowledge and attitudes regarding the assessment of pancreatitis in Saudi Arabia is limited. Therefore, the objective of this study is to fill this knowledge gap and enhance our understanding of this subject.

## Materials and methods

Study design 

This cross-sectional study aimed to assess the knowledge and awareness of pancreatitis risk factors and symptoms among the general population in Tabuk City, KSA. It was conducted as an observational study involving data collection from June 1, 2023, to August 30, 2023.

Study area

Tabuk City is located in the northwestern region of Saudi Arabia and has an approximate population of 534,893 people, according to recent data from Saudi Arabia's General Authority for Statistics.

Study population

The study included all adults aged 18 years or older residing in Tabuk City who willingly participated. Exclusions were made for individuals with pancreatic diseases or cognitive impairments.

Sample size

The sample size was determined using the following formula: 𝑛 = 𝑍²𝑝(1−𝑝) / 𝑑², where n = sample size, Z = confidence level (using a 1.96), p = 50% (to obtain the largest sample size, as the population distribution was not known), and d = 0.05 (margin of error). The calculation yielded 𝑛 = 1.96² x 0.5 (1-0.5) / 0.05² = 384; however, the sample size was increased to 549 to ensure the sample's representativeness.

Sampling technique

The sampling technique used for this study was random sampling. Participants were selected randomly from a variety of locations in community centers, including waiting areas at King Salman Hospital, King Fahad Specialist Hospital, King Khalid Hospital, Prince Sultan Hospital, and Primary Health Care Centers, Tabuk University, Prince Sultan University, Prince Fahad Bin Sultan Park, Tabuk Park Mall, Algarawi Centre, Alhokair Mall, Tabuk Boulevard, Grand Mall, Gallery Mall, Milagro Boutique, and AlSanabil Mall. This method aimed to ensure the sample's representativeness and sufficient participation.

Data collection tools

Data were collected through structured interviews using a questionnaire designed to fulfill the study's objectives and research questions (Appendix). The questionnaire, initially prepared in English, underwent rigorous review and revision by professionals. Data collectors were trained to translate questions into Arabic consistently. A pilot study with 25 participants not eligible for inclusion assessed the questionnaire's clarity and validity, resulting in a satisfactory Cronbach's alpha value of 0.74, indicating reliability.

Data analysis plan

The data was analyzed using IBM Corp. Released 2019. IBM SPSS Statistics for Windows, Version 26.0. Armonk, NY: IBM Corp. Descriptive statistics were employed to summarize the data. The chi-square test and logistic regression analysis were utilized to examine the relationship between knowledge and awareness of pancreatitis risk factors and symptoms and demographic characteristics. A test with a p-value of 0.05 or less was considered statistically significant.

Ethical consideration

The Institutional Review Board (IRB) of King Salman Armed Forces Hospital granted ethical permission for this study (approval number: KSAFH-REC-2023-512). Participants provided verbal informed consent before participation, and their responses were collected and processed confidentially to ensure optimal privacy.

## Results

The demographic information reveals that the total number of participants was 549, with an age range of 18 to above 60 years old. Females represented: n=260, 47.4%, while males accounted for n=289, 52.6% in Table 1.

**Table 1:** Sociodemographic information (n=549) N: Number of participants; %: Percentage of participants

Variables	Classifications	N	%
Age	18-30 years	350	63.8%
	31-45 years	82	14.9%
	46-60 years	95	17.3%
	>60 years	22	4.0%
Gender	Male	289	52.6%
	Female	260	47.4%
Marital Status	Single	292	53.2%
	Married	235	42.8%
	Divorced	11	2.0%
	Widowed	11	2.0%
Education Level	Elementary school	15	2.7%
	Middle school	15	2.7%
	High school	145	26.5%
	University	328	59.7%
	Higher education	46	8.4%
Occupation	Student	241	43.9%
	Employed	180	32.8%
	Unemployed	77	14.0%
	Retired	51	9.3%
Monthly income (SAR)	<5,000	192	35.0%
	5,000-10,000	89	16.2%
	10,001-15,000	30	5.5%
	15,001-20,000	63	11.5%
	>20,000	175	31.8%

According to Table 2, it is evident that the participants exhibited inadequate knowledge regarding the risk factors and symptoms of pancreatitis. Specifically, 508 participants (92.5%) reported never being diagnosed with pancreatitis, while 474 (86.3%) had no family history of pancreatitis. Additionally, 352 (64.1%) of the participants indicated that they had not received any information about pancreatitis and its risk factors.

**Table 2:** Knowledge about risk factors and symptoms of pancreatitis N: Number of participants; %: Percentage of participants

Question	Response	N	%
Have you ever been diagnosed with pancreatitis?	Yes	41	7.5%
	No	508	92.5%
Have you ever had gallstones?	Yes	51	9.3%
	No	498	90.7%
Have you ever consumed alcohol regularly?	Yes	18	3.3%
	No	531	96.7%
Have you ever had a history of high cholesterol?	Yes	111	20.2%
	No	438	79.8%
Have you ever had a family history of pancreatitis?	Yes	75	13.7%
	No	474	86.3%
Do you smoke?	Yes	116	21.1%
	No	433	78.9%
Do you experience any of the following symptoms? (Select all that apply)	Back pain	111	20.22%
	Nausea/vomiting,	147	26.78%
	I don't know	320	58.29%
	Jaundice	117	21.31%
	Unexplained weight loss	109	19.85%
	Upper abdominal pain	177	32.24%
Have you ever sought medical attention for any of the above symptoms?	Yes	158	28.8%
	No	391	71.2%
Have you ever received any information about pancreatitis and its risk factors?	Yes	197	35.9%
	No	352	64.1%
If you answered "yes" to the previous question, where did you receive this information? (Select all that apply)	Internet/online sources	128	23.32%
	from university	1	0.18%
	Healthcare provider	99	18.03%
	Family/friends	75	13.66%
	No answer	352	64.12%

As depicted in Table 3, we observed an inadequate response to the question: "Have you ever participated in any educational programs or campaigns regarding pancreatitis?" The majority indicated no participation (n=452, 82.3%). However, when it comes to the importance of increased awareness of pancreatitis and its risk factors for early detection and prevention, participants demonstrated adequate agreement (n=483, 88%). Moreover, when asked about their willingness to seek medical attention if they experienced any symptoms of pancreatitis, a significant number responded affirmatively (n=305, 55.6%). Regarding efforts to increase awareness about pancreatitis, most participants indicated that social media campaigns are the most effective tool (n=477, 86.89%).

**Table 3:** Awareness and education N: Number of participants; %: Percentage of participants

Question	Response	N	%
Have you ever participated in any educational programs or campaigns regarding pancreatitis?	Yes	97	17.7%
	No	452	82.3%
If you answered "yes" to the previous question, how did you participate? (Select all that apply)	No answer	452	82.33%
	Attended a seminar	50	9.11%
	Received educational materials (brochures, pamphlets, etc.	53	9.65%
	Watched a video	54	9.84%
	Lectures	1	0.18%
	Medicine collage	2	0.36%
Do you believe that increased awareness of pancreatitis and its risk factors would lead to early detection and prevention?	Yes	483	88.0%
	No	66	12.0%
How likely are you to seek medical attention if you experience any of the symptoms associated with pancreatitis?	Neutral	98	17.9%
	Somewhat likely	108	19.7%
	Somewhat unlikely	18	3.3%
	Very likely	305	55.6%
	Very unlikely	20	3.6%
In your opinion, what methods could be used to increase awareness about pancreatitis and its risk factors? (Select all that apply)	Educational seminars/workshops	216	39.34%
	Social media campaigns	477	86.89%
	TV/radio ads	227	41.35%
	Informational brochures/pamphlets	227	41.35%
	Short movies \ Animations	5	0.91%
	Word of mouth	5	0.91%

According to the results in Table 4, a highly significant association was observed between male and female responses. This association was evident in the following questions: "Do you experience any of the following symptoms?", "Have you ever had gallstones?" "Have you ever sought medical attention for any of the above symptoms?" and "How likely are you to seek medical attention if you experience any of the symptoms associated with pancreatitis?" (p = <0.0001, 0.007, 0.001, and 0.007, respectively). Additionally, a significant association was noticed in the question: "In your opinion, what methods could be used to increase awareness about pancreatitis and its risk factors?" (p=0.034). However, non-significant associations were observed with other variables.

**Table 4:** Association between knowledge and awareness of pancreatitis and the responses of the participants. ***p<0.001 is statistically significant. The chi-square test was computed. **p<0.01 is statistically significant. The chi-square test was computed. *p<0.05 is statistically significant. The chi-square test was computed. F is Fisher’s exact test.

Variables	Responses Of the participants	Female No. 260 (47.4%)	Male No. 289 (52.6%)	p-value
Have you ever been diagnosed with pancreatitis?	Yes	14 (5.4%)	27 (9.3%)	0.054(F)
	No	246 (94.6%)	262 (90.7%)	
Have you ever had gallstones?	Yes	33 (12.7%)	18 (6.2%)	0.007(F)
	No	227 (87.3%)	271 (93.8%)	
Have you ever consumed alcohol regularly?	Yes	11 (4.2%)	7 (2.4%)	0.172(F)
	No	249 (95.8%)	282 (97.6%)	
Have you ever had a history of high cholesterol?	Yes	50 (19.2%)	61 (21.1%)	0.330(F)
	No	210 (80.8%)	228 (78.9%)	
Have you ever had a family history of pancreatitis?	Yes	32 (12.3%)	43 (14.9%)	0.226(F)
	No	228 (87.7%)	246 (85.1%)	
Do you smoke?	Yes	47 (18.1%)	69 (23.9%)	0.059(F)
	No	213 (81.9%)	220 (76.1%)	
Do you experience any of the following symptoms?	Back pain	45 (17.31%)	66 (22.84%)	<0.0001***
	Nausea/vomiting,	78 (30.0%)	91 (31.49%)	
	I don't know	150 (57.69%)	167 (57.79%)	
	Jaundice	55 (21.15%)	56 (19.38%)	
	Unexplained weight loss	55 (21.15%)	53 (18.34%)	
	Upper abdominal pain	85 (32.69%)	94 (32.53%)	
Have you ever sought medical attention for any of the above symptoms?	Yes	92 (35.4%)	66 (22.8%)	0.001(F)
	No	168 (64.6%)	223 (77.2%)	
Have you ever received any information about pancreatitis and its risk factors?	Yes	87 (33.5%)	110 (38.1%)	0.151(F)
	No	173 (66.5%)	179 (61.9%)	
If you answered "yes" to the previous question, where did you receive this information?	Internet/online sources	51 (19.62%)	77 (26.64%)	0.349
	from university	0 (0%)	1 (0.35%)	
	Healthcare provider	45 (17.31%)	54 (18.69%)	
	Family/friends	52 (20.0%)	54 (18.69%)	
	No answer	173 (66.54%)	179 (61.94%)	
Have you ever participated in any educational programs or campaigns regarding pancreatitis?	Yes	41 (15.8%)	56 (19.4%)	0.160(F)
	No	219 (84.2%)	233 (80.6%)	
If you answered "yes" to the previous question, how did you participate?	No answer	219 (84.23%)	233 (80.62%)	0.207
	Attended a seminar	25 (9.62%)	25 (8.65%)	
	Received educational materials (brochures, pamphlets, etc.	20 (7.69%)	33 (11.42%)	
	Watched a video	24 (9.23%)	33 (11.42%)	
	Lectures	0 (0%)	1 (0.35%)	
	Medicine collage	1 (0.38%)	1 (0.35%)	
Do you believe that increased awareness of pancreatitis and its risk factors would lead to early detection and prevention?	Yes	233 (89.6%)	250 (86.5%)	0.162(F)
	No	27 (10.4%)	39 (13.5%)	
How likely are you to seek medical attention if you experience any of the symptoms associated with pancreatitis?	Neutral	35 (13.5%)	63 (21.8%)	0.007**
	Somewhat likely	64 (24.6%)	44 (15.2%)	
	Somewhat unlikely	8 (3.1%)	10 (3.5%)	
	Very likely	147 (56.5%)	158 (54.7%)	
	Very unlikely	6 (2.3%)	14 (4.8%)	
In your opinion, what methods could be used to increase awareness about pancreatitis and its risk factors?	Educational seminars/workshops	105 (40.38%)	111 (38.41%)	0.034*
	Social media campaigns	161 (61.38%)	181 (62.63%)	
	TV/radio ads	53 (20.38%)	55 (19.03%)	
	Informational brochures/pamphlets	85 (32.69%)	95 (32.87%)	
	Short movies \ Animations	0 (0%)	5 (1.73%)	
	Word of mouth	5 (1.92%)	0 (0%)	

## Discussion

Pancreatitis is an inflammatory disorder of the pancreas caused by factors such as gallstones and excessive alcohol use rather than infectious organisms. Complex gene-environment interactions may initiate or influence the development and progression of the illness. The commonly held belief that acute, recurrent, and chronic pancreatitis are distinct disease entities has given way to the notion of a disease continuum: 30% of acute pancreatitis patients may develop a chronic form, often with overlap with recurrent pancreatitis in the intervening years [1].

Acute pancreatitis is becoming more common in Saudi Arabia, with a prevalence rate of 23.5 cases per 100,000 people. Early diagnosis and treatment of pancreatitis are crucial to preventing significant complications [13].

Our study included 549 individuals aged 18 and above. Among them, 260 (47.4%) were females, while 289 (52.6%) were males. Chronic pancreatitis (CP) predominantly affects middle-aged individuals, whereas the risk of acute pancreatitis (AP) gradually increases with age [14]. Although pancreatitis is uncommon in individuals under the age of 20, pediatric patients are increasingly being diagnosed with the condition [15]. The male gender is associated with increased mortality, even though the prevalence of AP is the same in both men and women [16]. Furthermore, men are more likely to experience recurrent acute pancreatitis (RAP), which can lead to CP as it involves the repair of necrotic tissue with fibrotic tissue. Consequently, men develop CP at a higher rate than women, with 12 cases per 100,000 compared to 6 cases per 100,000, respectively [3,17,18]. 

Most of the participants demonstrated insufficient awareness regarding their participation in educational programs or campaigns related to pancreatitis (n=452, 82.3%). Additionally, they exhibited inadequate knowledge of the risk factors and symptoms of pancreatitis. These findings are consistent with a cross-sectional study conducted by Mehmood et al. in 2019, which revealed that most residents had a poorer understanding of diagnostic workup plans and actions compared to management strategies. Furthermore, as indicated by Mehmood et al. in 2019, the need for more educational programs is crucial to enhancing understanding of clinical practice standards for the early management of acute pancreatitis (AP) [19].

A highly significant association was observed between gender categories and the symptoms of pancreatitis. Furthermore, a significant relationship was identified between seeking medical attention and experiencing symptoms of pancreatitis. However, a non-significant difference was noted between males and females regarding the receipt of information about pancreatitis and its risk factors, where inadequate information was noticed. These findings contrast with a study conducted at Umm Al-Qura University in Saudi Arabia, which stated that "the majority of students have a good level of awareness compared to their level of knowledge" [13].

A study conducted by Patil SV et al. in 2018 supported our findings. They stated that "our findings suggest that students were not familiar with the basic interventional knowledge of acute pancreatitis, indicating a need for further investigation" [20]. The results from our participants indicate that the most effective way to increase awareness about pancreatitis and its risk factors is through social media campaigns, followed by educational seminars and workshops. Some other studies also support our findings, as demonstrated by Jastaniah S., et al. in 2022 [13]. However, it's important to note the limitations of this study, including the lack of knowledge about pancreatitis among the participants and the fact that it is a single-center study with a small sample size. To address these limitations, participants may benefit from more education about guidelines for the early detection and management of pancreatitis. This could be achieved by organizing educational lectures and campaigns to enhance awareness and knowledge about pancreatitis. Collaborating with healthcare professionals and organizations can provide valuable resources for these educational initiatives.

## Conclusions

Globally, substantial efforts are underway to enhance our understanding of pancreatitis. Timely diagnosis and intervention for acute pancreatitis are crucial in preventing a range of complications. The participants in this study demonstrated insufficient knowledge and awareness of pancreatitis. It is imperative to intensify efforts aimed at enhancing knowledge and awareness among the population in Tabuk City. This can be achieved through lectures in schools and universities, as well as through broader awareness campaigns. Research on pancreatitis is notably limited; therefore, additional studies should be initiated in various regions of the kingdom to augment understanding, awareness, and identification of risk factors related to pancreatitis.

## Appendices

**Table 5:** The questionnaires that were used in this study

Section A: Demographic Information	
Variables	Classifications
	18-30 years
	31-45 years
	46-60 years
	>60 years
Gender	Male
	Female
Marital Status	Single
	Married
	Divorced
	Widowed
Education Level	Elementary school
	Middle school
	High school
	University
	Higher education
Occupation	Student
	Employed
	Unemployed
	Retired
Monthly Income	<5,000
	5,000-10,000
	10,001-15,000
	15,001-20,000
	>20,000
Question	Response
Section B: Risk Factors and Symptoms of Pancreatitis	
Have you ever been diagnosed with pancreatitis?	Yes
	No
Have you ever had gallstones?	Yes
	No
Have you ever consumed alcohol regularly?	Yes
	No
Have you ever had a history of high cholesterol?	Yes
	No
Have you ever had a family history of pancreatitis?	Yes
	No
Do you smoke?	Yes
	No
Do you experience any of the following symptoms? (Select all that apply)	Back pain
	Nausea/vomiting
	I don't know
	Jaundice
	Unexplained weight loss
	Upper abdominal pain
Have you ever sought medical attention for any of the above symptoms?	Yes
	No
Have you ever received any information about pancreatitis and its risk factors?	Yes
	No
If you answered "yes" to the previous question, where did you receive this information? (Select all that apply)	Internet/online sources
	from university
	Healthcare provider
	Family/friends
	No answer
Section C: Awareness and Education	
Question	Response
Have you ever participated in any educational programs or campaigns regarding pancreatitis?	Yes
	No
If you answered "yes" to the previous question, how did you participate? (Select all that apply)	No answer
	Attended a seminar
	Received educational materials (brochures, pamphlets, etc.)
	Watched a video
	Lectures
	Medicine collage
Do you believe that increased awareness of pancreatitis and its risk factors would lead to early detection and prevention?	Yes
	No
How likely are you to seek medical attention if you experience any of the symptoms associated with pancreatitis?	Neutral
	Somewhat likely
	Somewhat unlikely
	Very likely
	Very unlikely
In your opinion, what methods could be used to increase awareness about pancreatitis and its risk factors? (Select all that apply)	Educational seminars/workshops
	Social media campaigns
	TV/radio ads
	Informational brochures/pamphlets
	Short movies/animations
	Word of mouth
